# miRNAs-Based Molecular Signature for *KRAS* Mutated and Wild Type Colorectal Cancer: An Explorative Study

**DOI:** 10.1155/2020/4927120

**Published:** 2020-06-23

**Authors:** Elena Milanesi, Maria Dobre, Alina Ioana Bucuroiu, Vlad Herlea, Teodora Ecaterina Manuc, Alessandro Salvi, Giuseppina De Petro, Mircea Manuc, Gabriel Becheanu

**Affiliations:** ^1^Victor Babes National Institute of Pathology, 050096 Bucharest, Romania; ^2^Carol Davila University of Medicine and Pharmacy, 050474 Bucharest, Romania; ^3^Fundeni Clinical Institute, 022328 Bucharest, Romania; ^4^Division of Biology and Genetics, Department of Molecular and Translational Medicine, University of Brescia, 25123 Brescia, Italy

## Abstract

microRNAs (miRNAs) have been proposed as promising molecular biomarkers for diagnosis, prognosis, and responsive therapeutic targets in different types of cancer, including colorectal cancer (CRC). In this study, we evaluated the expression levels of 84 cancer-associated miRNAs in a cohort of 39 human samples comprising 13 peritumoral and 26 tumoral tissues from surgical specimens of CRC patients. *KRAS* mutations were detected in 11 tumoral samples. In a first analysis, we found 5 miRNAs (miR-215-5p, miR-9-5p, miR-138-5p, miR378a-3p, and miR-150-5p) that were significantly downregulated and one upregulated (miR-135b-5p) in tumoral tissues compared with the peritumoral tissues. Furthermore, by comparing miRNA profile between *KRAS* mutated CRC tissues respect to wild type CRC tissues, we found 7 miRNA (miR-27b-3p, miR-191-5p, miR-let7d-5p, miR-15b-5p, miR-98-5p, miR-10a-5p, and miR-149-5p) downregulated in *KRAS* mutated condition. In conclusion, we have identified a panel of miRNAs that specifically distinguish CRC tissues from peritumoral tissue and a different set of miRNAs specific for CRC with *KRAS* mutations. These findings may contribute to the discovering of new molecular biomarkers with clinic relevance and might shed light on novel molecular aspects of CRC.

## 1. Introduction

According to GLOBOCAN 2018 (Global Cancer Observatory), colorectal cancer (CRC) is the second leading cause of cancer-related death worldwide and the fourth most incident cancer in the world, with a higher incidence among men [1]. Although advances in early detection and treatment options have reduced CRC mortality in developed nations, these countries remain those at the highest risk. 70-80% of cases of CRC occur sporadically and depend on risk factors that include history ulcerative colitis and Crohn's disease [2], but also constellation of modifiable environmental factors, more frequent in western countries, which include obesity, physical inactivity, poor diets, alcohol drinking, and smoking [3]. Approximately 25% of CRC patients have a positive family history of CRC, suggesting a specific contribution of inherited genetic factors [4]. Multiple whole-genome sequencing studies have been performed so far; however, only a small number of genetic variants have been successfully replicated in independent cohorts [5]. Moreover, it has been estimated that only 5%–10% of CRC are due to inherited mutations in well-known cancer-related genes [6]. Three canonical major distinct genetic pathways have been attributed to the development of sporadic CRC. These are not mutually exclusive and include the chromosomal instability pathway (CIN), the microsatellite instability pathway (MSI), and the CpG island methylator phenotype pathway (CIMP) [7]. The CIN pathway is the most frequent; it involves the classic adenoma-carcinoma sequence and genetic alterations in adenomatous polyposis coli—APC—(30–70%) and Kirsten Rat Sarcoma viral antigen homolog—*KRAS*—(30–50%). The CIMP pathway, reported in the 20-30% of sporadic CRC [8], involves the serrated neoplasia pathway and mutations in *KRAS* 10% (usually B-Raf proto-oncogene serine/threonine kinase—*BRAF*—wild type) and *BRAF*~70% [9]. The MSI pathway can involve both serrated neoplasia or adenoma-carcinoma sequence, which is characterized by mutations in *KRAS* 10%, *BRAF*~70% [10], and mutations in mismatch repair genes for Lynch syndrome.

In general, mutations of *KRAS* gene have been detected in approximately 40% of patients with CRC [11, 12]. These mutations are single nucleotide point variations and the most frequent are G12D, G12A, G12R, G12C, G12S, G12V, and G13D. In the codon 12, the mutations, G12D and G12V, are the most frequent, whereas in codon 13, the most frequent is G13D [13]. However, *KRAS* mutations also occur in codons 18, 61, 117, and 146, but at low frequencies compared with codons 12/13. The evaluation of *KRAS* mutation status in CRC patients has a crucial prognostic role, since patients carrying *KRAS* mutations have a poor response to anti-EGFR therapy [14, 15] and show an increased cumulative incidence of metastatic disease [16].

microRNAs are 19-22 nucleotide-long noncoding RNAs that regulate gene expression mainly at posttranscriptional level by binding to the 3′ untranslated region (3'UTR) of target mRNAs. Dysregulation of micro-RNAs expression levels has been observed in several human diseases, including cancers [17].

As the oncogene KRAS has been found upregulated in many human malignancies [18], the regulation of KRAS by miRNAs has drawn attention in the field, since specific miRNAs can act as tumor suppressor by targeting KRAS [19] also in CRC [20]. Indeed, even though miRNAs are not directly involved in mutagenesis mechanism nor modify the onset of mutations, they are key actors in inhibiting overexpressed mRNAs of genes harboring activating mutations such as *APC*, *TP53*, *KRAS*, and *BRAF* [21].

In this study, we aim to: (1) identify miRNAs differentially expressed between tumoral and peritumoral tissues from patients with CRC and (2) identify miRNAs differentially expressed in KRAS mutated patients versus Wild Type patients.

## 2. Materials and Methods

### 2.1. Collection of Human Tissue Samples

Twenty-six tumoral and thirteen corresponding peritumoral surgical specimens were collected from patients with primary CRC who underwent tumor surgical resection at “Fundeni” Clinical Institute in Bucharest, Romania. The specimens have been preserved in RNA later. The present study has been approved by the local ethics committee (registration number 291 of 8^th^ March 2016) and carried out in accordance with the Code of Ethics of the World Medical Association (Declaration of Helsinki). All the patients recruited have signed a written informed consent. All samples were examined by one experienced pathologist, and the socio-demographic and clinical information of the considered cohort are listed in Table 1.

### 2.2. KRAS Mutation Detection and miRNAs Expression Analysis

DNA has been isolated with QIAamp DNA Mini Kit (Qiagen, Germany). Total RNA, including miRNAs, has been isolated from RNA later preserved tissues using miRNeasy Mini Kit (Qiagen, Germany). Both isolations have been performed using the manufacturer's protocol. RNA and DNA quality and quantity were assessed by spectrophotometric method (NanoDrop 2000, Thermo Scientific) with both 260/280 nm and 260/230 nm parameters >1.8. KRAS mutations (in codons 12, 13, 61) were identified through pyrosequencing analysis using CEIVD marked PyroMark KRAS kit (QIAGEN, Hilden, Germany) according to the manufacturer's protocols on PyroMark Q24 instrument (QIAGEN, Hilden, Germany) and analyzed by Pyro Mark Q24 1.0.6.3 software as previously reported [22]. Reverse transcription of 500 ng of total RNA was performed with the miScript II RT Kit (Qiagen), and the expression of a panel including 84 miRNAs was evaluated with miScript™ miRNA PCR ArrayHuman Cancer PathwayFinder (MIHS-102Z, Qiagen) and miScript SYBR Green PCR Kit (Qiagen). This panel includes miRNAs previously correlated with the diagnosis, staging, progression, or prognosis of various cancers or tumors. Each array contains several control assays: six different snoRNA/snRNA as a normalization control for the array data (SNORD61, SNORD68, SNORD7, SNORD95, SNORD96A, RNU6B/RNU6-2), miRNA reverse transcription control (RTC) and positive PCR control (PPC). The miRNA expression was calculated by the 2−*Δ*CT method normalizing on the geometric mean of three controls (SNORD61, SNORD95, and SNORD96A) [23]. These three miRNAs have been chosen based on the RefFinder algorithm [24].

### 2.3. In Silico microRNA Target Identification

miRNA target identification has been performed using miRTarBase that comprises more than three hundred and sixty thousand miRNA-target interactions which are experimentally validated by reporter assay, western blot, microarray, and next-generation sequencing experiments [25]. A pathway analysis on the validated targets has been performed with KEGG through Enrichr, a comprehensive gene set enrichment analysis web server [26]. The analysis of the predicted oncogenes and tumor suppressors targeted by the selected miRNAs has been performed using miRWalk 2.0 (http://mirwalk.umm.uni-heidelberg.de/), and the total number of significantly enriched genes was calculated using Fisher's exact test (*p* < 0.05).

### 2.4. Statistical Analysis

Categorical variables were tested by means of the chi-square test and continuous variables with the *t*-test. The normality of data distribution of each miRNA level was evaluated using the Shapiro–Wilk test. Since data were not normally distributed, differences in miRNA expression between Tumoral and Peritumoral tissues were assessed using the Mann–Whitney *U* test. A further analysis for the 13 tumoral samples with the matched peritumoral tissues has been performed using a paired-sample *t*-test. miRNAs expression differences among the three groups were evaluated using the nonparametric Kruskal–Wallis test followed by pairwise tests. miRNA levels changes were considered significant between the groups when the *p* value was <0.05 and the fold regulation (FR) was FR >2 or FR <-2. Statistical analysis was performed using the Statistical Package for Social Science (SPSS version 17.0).

## 3. Results

**Figure 1:**
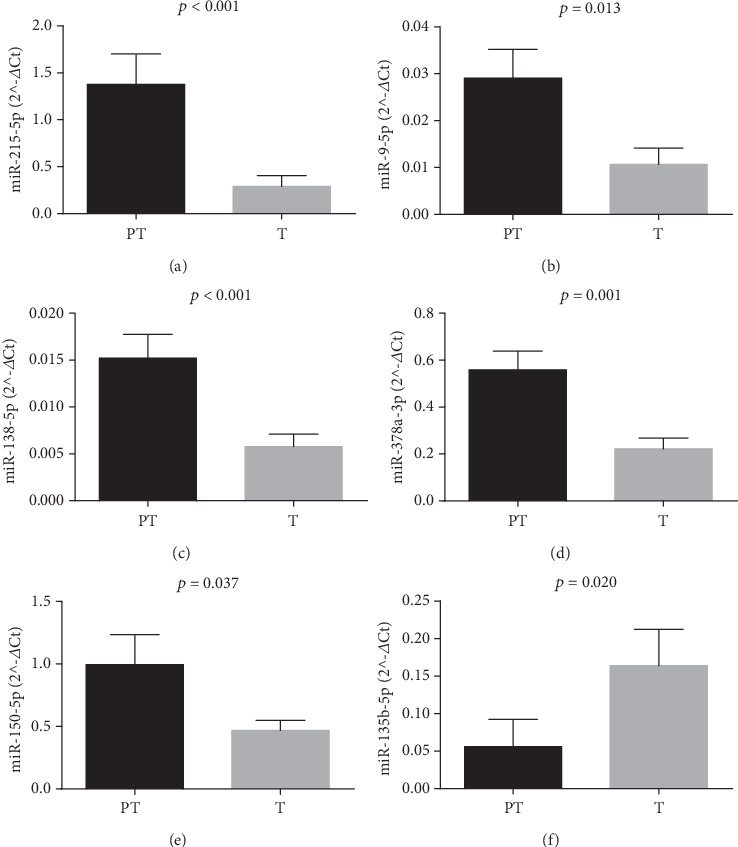
The graphs show the significant miRNAs differentially expressed between tumoral and peritumoral tissues. Bar graphs represent the mean of the 2^−*ΔCt*^ values, and error bars represent the standard error. *p* values have been calculated using the Mann–Whitney *U* test. (a) miR-215-5p; (b) miR-19-5p; (c) miR-138-5p; (d) miR-378a-5p; (e) miR-150-5p; (f) miR-135b-5p. PT: peritumoral; T: tumoral.

In this study, we evaluated the expression of 84 cancer-associated miRNAs known to play a pivotal role in tumor onset and progression. The general expression of each miRNA in terms of Ct range is shown in Supplementary data (Table S1). In a first analysis, we compared the miRNAs expression profile between peritumoral and tumoral CRC tissues from surgical specimens (including those with and without *KRAS* mutations). *KRAS* mutations were not identified in peritumoral tissues. The two groups were homogenous for age and sex. We found six miRNAs differentially expressed, five downregulated and one upregulated in tumoral tissues compared to peritumoral tissues (Table 2). The graphic representation of the significant miRNAs is shown in Figure 1. We further performed a paired analysis for the 13 tumoral samples with the matched peritumoral tissues. The results showed that miR-215-5p was significantly downregulated also in the small group (FR = −2.87, *p* = 0.003). miR-9-5p, miR-138-5p, miR-378a-3p, miR-150-5p, and miR-135b-5p maintained the same trend of expression obtained considering all the cases, with a fold regulation of -1.49, -1.92, -1.73, -1.77, and +3.82, respectively. However, for these miRNAs, the statistical significance was lost.

**Figure 2:**
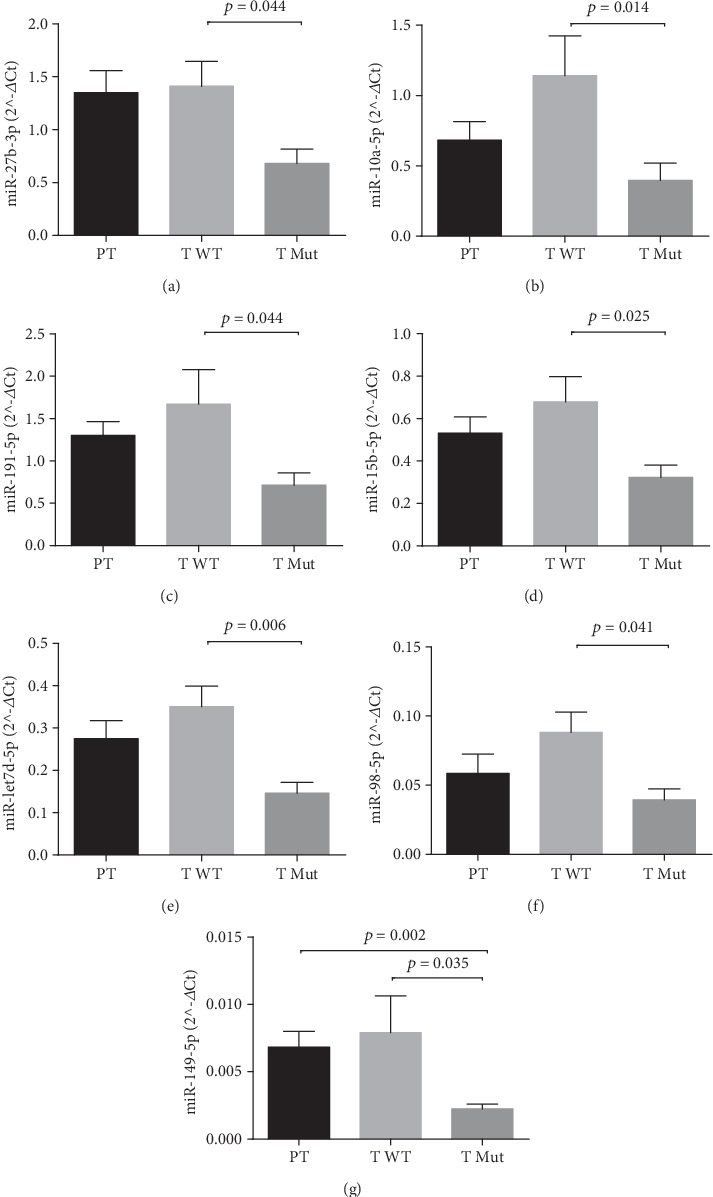
(a–g) The graphs show the significant miRNAs differentially expressed among the groups (PT: peritumoral; T WT: tumoral KRAS wild type; T Mut: tumoral KRAS mutated). Bar graphs represent the mean of the 2^−*Δ*Ct^ values, and error bars represent the standard error. (a) miR-27b-3p. (b) miR- 10a-5p. (c) miR-191-5p. (d) miR-15b-5p. (e) miR-let7d-5p. (f) miR-98-5p. (g) miR- 149-5p. *p* value has been calculated using Kruskal–Wallis test followed by pairwise tests.

In a second analysis, we focused on the differences of miRNA profile between wild type and *KRAS* mutated tumoral tissues performing a comparison between the two groups. We found that 7 miRNAs were downregulated in patients carrying *KRAS* mutations compared to wild type patients. These results are reported in Table 3 and in Figure 2.

### 3.1. microRNA Target Identification and Pathway Analysis

The identification of the mRNA targets has been performed for the most significant miRNAs up- and downregulated in the comparison between tumoral and peritumoral tissues, miR-135b-5p and miR-215-5p, respectively, and for the most downregulated miRNA in tumoral tissues with KRAS mutations vs tumoral wild type tissues (miR-27b-3p). Only experimentally validated targets have been considered. Among the validated targets, we reported those directly involved in CRC pathway according to KEGG pathway analysis (Table 4). We have also reported the total number of the significantly enriched oncogenes and tumor suppressors predicted to be targeted by the selected miRNAs using miRWalk.

## 4. Discussion

In this exploratory study, we analyzed a cohort of 39 samples representing 13 peritumoral and 26 tumoral tissues from surgical specimens of CRC patients, in order to identify a specific miRNAs molecular signature of CRC able to discriminate PT tissues from CRC tissues and CRC *KRAS* mutated tissues from CRC wild type tissues, by analyzing 84 candidate miRNAs by qPCR array. This analysis identified 5 miRNA (miR-215-5p, miR-9-5p, miR-138-5p, miR378a-3p, and miR-150-5p) that were significantly downregulated and one upregulated (miR-135b-5p) in tumoral tissues compared with the peritumoral control group. We further stratified the tumoral tissues according to the presence (T_M) or lack (T_WT) of *KRAS* mutations, and we compared the miRNAs profile of the two groups to assess the miRNAs differentially expressed in CRC mutated respect to CRC wild type. We identified 7 miRNAs (miR-27b-3p, miR-191-5p, miR-let7d-5p, miR-10a-5p, miR-15b-5p, miR-98-5p, and miR-149-5p) all downregulated in *KRAS* mutation carriers compared to the wild type patients.

Consistent with our findings, most of the miRNAs identified in the first comparison have been previously reported to be significantly dysregulated in CRC, and they play important roles in tumor development. Recently, Falzone and collaborators performed an integrated analysis of 10 miRNAs datasets carrying out a bioinformatics analysis on 703 samples (262 normal tissues and 441 samples of colorectal carcinoma) [18]. They identified 20 significant differentially expressed miRNAs (10 downregulated and 10 upregulated) between colorectal cancer samples and normal tissues in at least 3 of 10 datasets. Among these miRNAs, in line with our findings, the authors identified miR-135b-5p (upregulated), and miR-378-3p, miR-150-5p, miR-215-5p (downregulated). The miR-215-5p has been predicted to target CXCL2 in CRC cell lines (HT29) [27]. Interestingly, CXCL2 has been found upregulated in inflamed mucosa compared to not inflamed mucosa of patients with ulcerative colitis [28], a condition that increases the risk of CRC. Another study demonstrated that miR-138-5p was significantly downregulated in CRC tissue samples and cell lines and showed that its overexpression delayed cell proliferation, reduced colony formation, and increased apoptosis in CRC cell lines [29, 30]. No studies showing differential expression of miR-9-5p between peritumoral and tumoral tissues are reported. However, miR-9-5p has been indicated a prognostic biomarker in CRC [31, 32].

Regarding the seven miRNAs, we found downregulated in *KRAS* mutation carriers compared to the WT, they have been all found implicated in CRC onset and progression, but no study has directly linked them to *KRAS* mutations. miR-27b-3p promoted migration and invasion in colorectal cancer cells by targeting HOXA10/integrin *β*1 cell signal axis [33]. A decrease of its levels has been observed in oxaliplatin-resistant cell lines suggesting this miRNA as valuable therapeutic target for CRC, especially for patients with chemoresistance [34]. Moreover, miR-27b-3p has also been found associated with other types of cancer showing a significant downregulation in gastric cancer cell lines and tissues compared with the normal group [35].

A single study associated a dysregulation of miR-191-5p in colon adenocarcinoma, suggesting this miRNA as possible prognostic marker [36], miR-191-5p has a relevant role in other types of cancer, including renal cell carcinoma [37] and osteosarcoma [38]. miRNAs let-7 family members generally promote differentiation during development and function as tumor suppressors in various cancers [39], and *Let-7d* regulation of *KRAS* has previously been shown [40]. Recent data indicated that let-7d-5p increases sensitivity to trifluridine, a key component of the antitumor drug trifluridine/tipiracil for the treatment of patients with metastatic colorectal cancer refractory to standard chemotherapies, suggesting this miRNA as a potential clinical marker of treatment sensitivity [41]. Moreover, miR-let-7d-5p was found upregulated in paraffin-embedded (FFPE) tissue samples of CRC patients compared to controls [42], without any data being reported in *KRAS* mutated samples. Recently, an *in situ* hybridization array approach, using paraffin-embedded biopsies of colorectal primary tumors, studied the expression levels of 1436 miRNAs in 192 samples. The miRNA profile has been associated with clinical and histopathological features indicating that miR-10a-5p is correlated with relevant histopathological features, including stroma abundance, tumor grade, peritumoral inflammatory infiltrates, mucin type, and tumor location [43]. Moreover, this miRNA was found associated with tumor localization being less abundant in the right colon compared to the left colon and rectum [44].

MiR-15b-5p was associated with different types of cancer, such as ovarian cancer [45], liver cancer [46], neuroblastoma [47]. In CRC, this miRNA has been suggested as potential therapeutic target for CRC treatment, particularly for 5-FU-resistant CRC [48] and potential target for metastatic CRC therapy [49]. Also, its levels could be useful to distinguish between CRC or its precancerous lesion (advanced adenomas) and healthy individuals controls [50]. MiR-98-5p levels have been found dysregulated in different types of cancer cells, such as nonsmall cell lung carcinoma [51], prostate cancer [52], and breast cancer [53]. In relation to CRC, this miRNA has been identified in human colon carcinoma cell line LIM1863--shed microvesicles [54]. Moreover, this miRNA is a member of a panel of six miRNAs that seem to predict treatment response to fluoropyrimidine containing first-line systemic treatment in patients with mCRC when combined with four clinicopathological factors [55]. In our study, miR-149-5p resulted downmodulated in *KRAS* mutated samples vs wild type; it has been demonstrated that LncRNA PCAT-1 regulated cell proliferation, invasion, migration, and apoptosis in colorectal cancer through targeting miR-149-5p [56], and according to our results, miR-149-5p resulted downregulated in CRC, likely acting as a tumor suppressor in CRC [57].

Clearly, there are some limitations for this study. Firstly, data regarding cancer evolution are not yet available, so the prognostic value of the identified miRNAs cannot be assessed. Secondly, another limitation is the relatively small sample size of the subgroup of the *KRAS* mutated samples.

## 5. Conclusions

In conclusion, the novelty of our work is the identification of a panel of miRNAs that resulted in dysregulated in CRC tissues compared to their normal adjacent tissues. The specific identification of a different set of miRNAs (all downregulated) in *KRAS* mutated CRC tissues respect to wild type CRC tissues could suggest their putative role as responsive molecular targets (i.e., by ectopically modifying their expression levels). These data could help to identify novel strategies to improve the efficacy of the therapy, mainly in the subgroup of patients with *KRAS* mutations. More studies and a wide cohort are needed to support the conclusions of our explorative study.

## Figures and Tables

**Table 1:** Clinical and pathological characteristics of CRC patients involved in the study.

	Tumor *KRAS* Mut (*N* = 11)	Tumor WT (*N* = 15)	Peritumoral (*N* = 13)	^#^ *p* value
Age	63.72 ± 8.12	64 ± 9.81	61.23 ± 8.96	T_WT vs PT = 0.445
				T_M vs PT = 0.486
				T_M vs T_WT = 0.941

Sex (%F)	27%	60.00%	53.80%	T_WT vs PT = 0.743 (*χ*2 = 0.108)
				T_M vs PT = 0.188 (*χ*2 = 1.731)
				T_M vs T_WT = 0.098 (*χ*2 = 2.735)

Tumor location	Colon 81.82%	Colon 60%	Colon 61.54%	∗RSJ: rectosigmoid junction ^#^*p* value for age was calculated using the *t*-test, whereas sex was tested by means of the chi-square test.
	Sigmoid 0%	Sigmoid 26.66%	Sigmoid 15.38%	
	RSJ∗ 9.09%	RSJ∗ 0%	RSJ∗ 7.70%	
	Rectum 9.09%	Rectum 13.34%	Rectum 15.38%	
TNM staging	T2N0M0 (*n* = 5) T2N1M0 (*n* = 0) T3N1M0 (*n* = 3) T3N2M0 (*n* = 1) T4N0M0 (*n* = 1) T4N1M0 (*n* = 1)	T2N0M0 (*n* = 4) T2N1M0 (*n* = 3) T3N1M0 (*n* = 5) T3N2M0 (*n* = 2) T4N0M0 (*n* = 0) T4N1M0 (*n* = 1)		
*KRAS* mutation	Codon 12 G12A (*n* = 1) G12C (*n* = 1) G12D (*n* = 1) G12R (*n* = 1) G12V (*n* = 2)			
	Codon 13 G13D (*n* = 3)			
	Codon 61 Q61E (*n* = 1) Q61L (*n* = 1)			

**Table 2:** miRNAs differentially expressed in tumoral (*n* = 26) vs peritumoral (*n* = 13) tissues. miRNAs are ordered accordingly to increasing fold regulation.

miRNA differentially expressed (26 T vs 13 PT)		
miRNAs	*p* value^#^	FR^∗^
miR-215-5p	<0.001	-4.75
miR-9-5p	0.013	-2.73
miR-138-5p	<0.001	-2.63
miR-378a-3p	0.001	-2.52
miR-150-5p	0.037	-2.12
miR-135b-5p	0.020	2.92

#*p*_value has been calculated using the Mann–Whitney *U* test; ∗ FR: fold regulation.

**Table 3:** The table shows the miRNAs differentially expressed among the three groups. In blue font, it is reported that the FR of the miRNAs downregulated in tumoral tissues with *KRAS* mutations vs tumoral *KRAS* wild type.

miRNA	*p* value KW^#^	Pairwise comparison	Adjusted *p* value pairwise	FR^∗^
miR-27b-3p	0.032	T_WT vs PT	0.999	1.04
		T_M vs PT	0.088	-1.98
		T_M vs T_WT	0.044	-2.07

miR-191-5p	0.032	T_WT vs PT	0.999	1.28
		T_M vs PT	0.088	-1.82
		T_M vs T_WT	0.044	-2.33

miR-let7d-5p	0.007	T_WT vs PT	0.999	1.27
		T_M vs PT	0.087	-1.88
		T_M vs T_WT	0.006	-2.40

miR-10a-5p	0.017	T_WT vs PT	0.999	1.67
		T_M vs PT	0.169	-1.73
		T_M vs T_WT	0.014	-2.88

miR-15b-5p	0.027	T_WT vs PT	0.999	1.28
		T_M vs PT	0.174	-1.65
		T_M vs T_WT	0.025	-2.10

miR-98-5p	0.038	T_WT vs PT	0.264	1.51
		T_M vs PT	0.999	-1.49
		T_M vs T_WT	0.041	-2.24

miR-149-5p	0.002	T_WT vs PT	0.848	1.16
		T_M vs PT	0.002	-3.03
		T_M vs T_WT	0.035	-3.51

^#^KW: Kruskal–Wallis test followed by pairwise tests. ^∗^FR: fold regulation.

**Table 4:** In the table, data are reported for each miRNA: the number of the validated targets according to miRTarbase; the validated targets belonging to CRC pathway according to KEGG, the number of predicted oncogene and tumor suppressor genes targets according to miRWalk analysis.

miRNAs	N. of validated targets (miRTarbase)	Validated targets in CRC pathway (KEGG)	N. of predicted targets OG (miRWalk)	N. of predicted targets TSG (miRWalk)
miR-215-5p	754	APC, APPL1, BCL2, BCL2L11, CYCS, FOS, MAPK9, MSH6, RPS6KB1, TCF7 (*p* = 0.0014; *adj* − *p* = 0.06)	134 (*p* value <0.0001)	26 (*p* value = 0.07)
miR-135b-5p	84	APC, BIRC5, MYC, TGFBR1 (*p* = 0.0004; *adj* − *p* = 0.03481)	207 (*p* value <0.0001)	45 (*p* value = 0.009)
miR-27b-3p	426	EGFR, MAPK9, NRAS, PMAIP1, SMAD2, SOS1, TGFBR1 (*p* = 0.0004; *adj* − *p* = 0.009)	227 (*p* value <0.0001)	49 (*p* value = 0.051)

OG: oncogenes; TSG: tumor suppressor gene. *p* value has been calculated using the Fisher exact test.

## Data Availability

The data used to support the findings of this study are included within the article and in the Supplementary Materials.

## Supplementary Materials

Supplementary Materials Table S1: gene expression range of all the 84 miRNAs measured in tumoral CRC and peritumoral samples. The expression is reported as qPCR Ct range value.
