# Supplementary material for: miRNAs-Based Molecular Signature for KRAS Mutated and Wild Type Colorectal Cancer: An Explorative Study
Source: J Immunol Res. 2020 Jun 23;2020:4927120. doi: 10.1155/2020/4927120 (PMC7330647; doi:10.1155/2020/4927120)
Supplement: Supplementary Materials — Table S1: gene expression range of all the 84 miRNAs measured in tumoral CRC and peritumoral samples. The expression is reported as qPCR Ct range value. [file 4927120.f1.docx]

Supplementary Data

|  | **miRNA** | **Ct range** |  | **miRNA** | **Ct range** |
| --- | --- | --- | --- | --- | --- |
| 1 | hsa-let-7a-5p | 20-25 | 43 | hsa-miR-181d | 25-30 |
| 2 | hsa-miR-133b | 25-30 | 44 | hsa-miR-301a-3p | 25-30 |
| 3 | hsa-miR-122-5p | 30-34 | 45 | hsa-miR-200c-3p | <20 |
| 4 | hsa-miR-20b-5p | 20-25 | 46 | hsa-miR-100-5p | <20 |
| 5 | hsa-miR-335-5p | 25-30 | 47 | hsa-miR-10b-5p | 20-25 |
| 6 | hsa-miR-196a-5p | 20-25 | 48 | hsa-miR-155-5p | 20-25 |
| 7 | hsa-miR-125a-5p | 20-25 | 49 | hsa-miR-1 | 20-25 |
| 8 | hsa-miR-142-5p | 20-25 | 50 | hsa-miR-150-5p | 20-25 |
| 9 | hsa-miR-96-5p | 25-30 | 51 | hsa-let-7i-5p | 20-25 |
| 10 | hsa-miR-222-3p | 20-25 | 52 | hsa-miR-27b-3p | <20 |
| 11 | hsa-miR-148b-3p | 20-25 | 53 | hsa-miR-7-5p | 20-25 |
| 12 | hsa-miR-92a-3p | 20-25 | 54 | hsa-miR-127-5p | 25-30 |
| 13 | hsa-miR-184 | ≥35 | 55 | hsa-miR-29a-3p | <20 |
| 14 | hsa-miR-214-3p | 20-25 | 56 | hsa-miR-191-5p | <20 |
| 15 | hsa-miR-15a-5p | 20-25 | 57 | hsa-let-7d-5p | 20-25 |
| 16 | hsa-miR-378a-3p | 20-25 | 58 | hsa-miR-9-5p | 25-30 |
| 17 | hsa-let-7b-5p | <20 | 59 | hsa-let-7f-5p | 20-25 |
| 18 | hsa-miR-205-5p | 25-30 | 60 | hsa-miR-10a-5p | 20-25 |
| 19 | hsa-miR-181a-5p | 20-25 | 61 | hsa-miR-181b-5p | 20-25 |
| 20 | hsa-miR-130a-3p | 20-25 | 62 | hsa-miR-15b-5p | 20-25 |
| 21 | hsa-miR-140-5p | 20-25 | 63 | hsa-miR-16-5p | <20 |
| 22 | hsa-miR-20a-5p | <20 | 64 | hsa-miR-210 | 20-25 |
| 23 | hsa-miR-146b-5p | 20-25 | 65 | hsa-miR-17-5p | <20 |
| 24 | hsa-miR-132-3p | 25-30 | 66 | hsa-miR-98-5p | 20-25 |
| 25 | hsa-miR-193b-3p | 20-25 | 67 | hsa-miR-34a-5p | 20-25 |
| 26 | hsa-miR-183-5p | 25-30 | 68 | hsa-miR-25-3p | 20-25 |
| 27 | hsa-miR-34c-5p | 25-30 | 69 | hsa-miR-144-3p | 25-30 |
| 28 | hsa-miR-30c-5p | 20-25 | 70 | hsa-miR-128 | 20-25 |
| 29 | hsa-miR-148a-3p | <20 | 71 | hsa-miR-143-3p | <20 |
| 30 | hsa-miR-134 | 25-30 | 72 | hsa-miR-215 | 20-25 |
| 31 | hsa-let-7g-5p | 20-25 | 73 | hsa-miR-19a-3p | <20 |
| 32 | hsa-miR-138-5p | 25-30 | 74 | hsa-miR-193a-5p | 20-25 |
| 33 | hsa-miR-373-3p | 30-34 | 75 | hsa-miR-18a-5p | 20-25 |
| 34 | hsa-let-7c | 20-25 | 76 | hsa-miR-125b-5p | <20 |
| 35 | hsa-let-7e-5p | 20-25 | 77 | hsa-miR-126-3p | <20 |
| 36 | hsa-miR-218-5p | 25-30 | 78 | hsa-miR-27a-3p | <20 |
| 37 | hsa-miR-29b-3p | <20 | 79 | hsa-miR-372 | 30-34 |
| 38 | hsa-miR-146a-5p | 20-25 | 80 | hsa-miR-149-5p | 25-30 |
| 39 | hsa-miR-135b-5p | 25-30 | 81 | hsa-miR-23b-3p | <20 |
| 40 | hsa-miR-206 | 30-34 | 82 | hsa-miR-203a | 20-25 |
| 41 | hsa-miR-124-3p | 25-30 | 83 | hsa-miR-32-5p | 25-30 |
| 42 | hsa-miR-21-5p | <20 | 84 | hsa-miR-181c-5p | 20-25 |
|  |  |  |  |  |  |

Table S1: Gene expression range of all the 84 miRNAs measured in tumoral CRC and peritumoral samples. The expression is reported as qPCR Ct range value.
